# Recent Advances in the Applications of Small Molecules in the Treatment of Multiple Myeloma

**DOI:** 10.3390/ijms24032645

**Published:** 2023-01-31

**Authors:** Hanley N. Abramson

**Affiliations:** Department of Pharmaceutical Sciences, Eugene Applebaum College of Pharmacy and Health Sciences, Wayne State University, Detroit, MI 48202, USA; ac2531@wayne.edu

**Keywords:** myeloma, melflufen, cereblon E3 ligase modulators, venetoclax, selinexor

## Abstract

Therapy for multiple myeloma (MM), a hematologic neoplasm of plasma cells, has undergone remarkable changes over the past 25 years. Small molecules (molecular weight of less than one kDa), together with newer immunotherapies that include monoclonal antibodies, antibody-drug conjugates, and most recently, chimeric antigen receptor (CAR) T-cells, have combined to double the disease’s five-year survival rate to over 50% during the past few decades. Despite these advances, the disease is still considered incurable, and its treatment continues to pose substantial challenges, since therapeutic refractoriness and patient relapse are exceedingly common. This review focuses on the current pipeline, along with the contemporary roles and future prospects for small molecules in MM therapy. While small molecules offer prospective benefits in terms of oral bioavailability, cellular penetration, simplicity of preparation, and improved cost–benefit considerations, they also pose problems of toxicity due to off-target effects. Highlighted in the discussion are recent developments in the applications of alkylating agents, immunomodulators, proteasome inhibitors, apoptosis inducers, kinesin spindle protein inhibitors, blockers of nuclear transport, and drugs that affect various kinases involved in intracellular signaling pathways. Molecular and cellular targets are described for each class of agents in relation to their roles as drivers of MM.

## 1. Introduction

Multiple myeloma (MM) is a hematologic malignancy in which plasma cells proliferate abnormally in the bone marrow, resulting in excessive levels of monoclonal immunoglobulins in urine and/or blood. The disease typically is accompanied by hyper***c***alcemia, ***r***enal insufficiency, ***a***nemia, and ***b***one pain (the so-called CRAB tetrad of symptoms), as well as propensity to infection. MM ranks 14th in incidence among all cancers and second to non-Hodgkin’s lymphoma (NHL) as a hematologic cancer [1]. According to current estimates, in 2022 MM will be diagnosed in a total of 34,470 individuals (55.4% male) and will be responsible for 12,640 (56.1% male) deaths in the U.S. [2]. The median age at diagnosis is 69 in the U.S. [3]. Globally, in 2020 there were an estimated 176,404 cases of the disease (0.9% of all cancers), accounting for 117,077 deaths (1.2% of cancer deaths) [4]. Substantial racial disparities have been noted for all stages of MM. According to available data, compared to Caucasians, the incidence rates for the disease are higher by 2.1 times in African American men and 2.6 times in African American women [5]. Updated guidelines for the diagnosis and treatment of MM are published annually by the National Comprehensive Cancer Network (NCCN) [6]. Although the cause of MM remains unknown, cytogenetic factors are known to play a role in some MM patients classified as “high risk”. Among the most frequently encountered of these anomalies are the chromosomal deletion del(17p) and the transversions t(14;16) and t(4;14) [7].

Starting in the 1950s, the standard treatment for MM consisted of alkylating agents (melphalan and/or cyclophosphamide), often in combination with corticosteroids. Autologous stem cell transplantation (ASCT) was added to the regimen beginning in the mid-1980s. Discovery of the remarkable anti-myeloma actions of thalidomide in 1999 signaled the start of a revolutionary change in the therapeutic approach to the disease as related compounds, such as lenalidomide and pomalidomide, became available soon afterward. The therapeutic landscape for MM broadened further during the first two decades of the current century with the discovery of entirely new drug classes effective against the disease: proteasome inhibitors (bortezomib), histone deacetylase inhibitors (panobinostat), and more recently, nuclear export inhibitors (selinexor). Introduction of immunotherapy as a viable approach to MM treatment began in 2015 with the approval of the monoclonal antibodies daratumumab (anti-CD38) and elotuzumab (anti-SLAMF7) and continued into the present decade with the antibody-drug conjugate belantamab mafodotin, the bispecific teclistamab, and chimeric antibody receptor (CAR) T-cell products, such as idecabtagene vicleucel. The cumulative effect of these and related advances has raised the five-year survival rate for MM to 55% for patients diagnosed with the disease in the period 2011–2017, compared to the situation in the mid-1970s when only a quarter of those diagnosed with MM survived five years [8]. Although the advances made thus far concerning MM have been outstanding, the fact remains that MM still is considered incurable, and although treatment measures may be initially successful, patients nearly always become refractory to therapy and relapse is the result.

The successes of the past quarter-century notwithstanding, the search for new drugs to treat relapsed and/or refractory MM (RRMM) remains a high priority in the field of drug design and development. The trend toward immunologic approaches to the disease, has been complemented by the continued emphasis accorded to “small molecules”, i.e., those with molecular weights generally lower than about one kDa. The latter offer the potential for ease of cellular entry, oral effectiveness, and comparative simplicity of synthetic preparation, as well for improved cost-benefit analyses when used with immune-based drugs [9,10].

Although the design of small molecules intended to interfere with the interactions of far larger macromolecules, such as nucleic acid sequences or protein domains, poses substantial problems, the field of anti-cancer drug design abounds with successes in this regard, such as the development of orally effective inhibitors of tyrosine kinase, cyclin-dependent kinases, and cereblon E3 ligase. The present review is intended to provide a synopsis of the current state of small molecules intended to treat MM, with a focus on those agents currently included in active clinical trials but not yet approved by the U.S. Food and Drug Administration (FDA). The discussion is organized primarily according to the mechanisms of action or molecular/cellular targets of the included drugs.

## 2. Alkylating Agents

**Figure 1:**
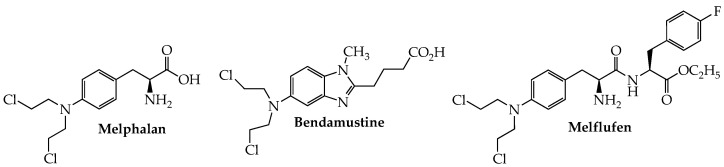
Structures of Alkylating Agents with Activity Against Multiple Myeloma.

The use of alkylating agents (Figure 1) in MM dates back to the late 1950s with the first reports of melphalan (Alkeran, sarcolysin, phenylalanine mustard) in the treatment of this cancer [11]. Although this picture began to change dramatically early in the current century with the introduction of anti-myeloma drugs working by other mechanisms, interest in developing new alkylating agents to treat MM has not entirely disappeared. One such example is bendamustine, which contains a reactive mustard functionality linked to a purine-like benzimidazole ring and has been approved by the FDA for chronic lymphocytic leukemia (CLL) and indolent B-cell NHL. Although this hybrid molecule is not approved in the U.S. for MM, it is licensed in Europe, under the trade name Levact, for newly diagnosed MM (NDMM).

Overexpression of aminopeptidases in MM provides the basis for the design of melflufen (melphalan flufenamide; Pepaxto), a dipeptide in which melphalan is linked to p-fluorophenylalanine ethyl ester. The highly lipophilic nature of the conjugate enables its ready passage through the cell membrane; subsequent intracellular cleavage by aminopeptidase N (CD13) releases the active alkylator, in concentrations higher than when melphalan is given alone, to cause irreversible DNA damage and consequent apoptosis in myeloma cells, especially those resistant to melphalan [12]. Melflufen in combination with dexamethasone was granted accelerated approval by the FDA in Feb. 2021 for RRMM in patients who have previously failed at least four lines of therapy, including a proteasome inhibitor, anti-CD38 mAb, and an immunomodulator [13,14]. Efficacy [ORR = 23.7%; median duration of response (DOR) = 4.2 months] and safety data provided in the HORIZON trial (NCT02963493) served as the primary basis for the approval. Analysis of data from a phase III confirmatory study (NCT03151811; OCEAN) in which RRMM patients received melflufen and pomalidomide plus dexamethasone found that the melflufen group showed superior progression-free survival (PFS) compared to the pomalidomide cohort (6.8 vs. 4.9 months) [15]. However, examination of OS data from this trial found a higher death rate (48% vs. 43%) in the melflufen group, leading the FDA in October 2021 to withdraw its approval of melflufen for use in RRMM [16].

## 3. Cereblon E3 Ligase Modulators

Immunomodulation as a viable treatment approach for MM owes its origin to thalidomide, first approved by the FDA for the disease in 2006. Lenalidomide (Revlimid) (2006) and pomalidomide (Pomalyst) (2015), two related derivatives of thalidomide, subsequently were approved for use in the disease. The mechanistic basis for the anti-tumor properties of this drug class has been tracked to their ability to bind to cereblon, which in turn is enabled to form a complex with three other proteins: CUL4 (cullin4), Roc1 (regulator of cullins 1), and DDB1 (DNA damage-binding protein 1), which collectively constitute cullin-4 RING E3 ligase (CRL4), an enzyme with E3 ubiquitin ligase activity. Two zinc finger transcription factors, Ikaros (IKZF1) and Aiolos (IKZF3), which are essential for myeloma cell survival, are recruited to the complex as neo-substrates, resulting in their ubiquitination and subsequent proteasomal degradation [17,18]. This capacity of small molecules (known as degraders or “molecular glues”), such as the thalidomide-related anti-myeloma immunomodulators (IMiDs), to hijack the specificity of E3 ubiquitin ligases to bring about targeted degradation of disease-linked proteins, in fact, has helped usher in the field of targeted protein degradation (TPD) as an entirely new and stimulating concept in drug discovery [19,20,21]. A related strategy known as proteolysis-targeting chimera (PROTAC), which generally employs a small molecule linker that bridges an E3 ubiquitin ligase and a protein targeted for degradation, represents an extension of this approach also with potential application to cancer treatment [22].

According to current NCCN guidelines [6], lenalidomide plus dexamethasone in combination with either bortezomib or daratumumab is considered the preferred primary regimen for both transplant-eligible and -ineligible patients. Interestingly, several studies have demonstrated the efficacy of pomalidomide in about one-third of myeloma patients refractory to lenalidomide [23,24,25,26].

**Figure 2:**
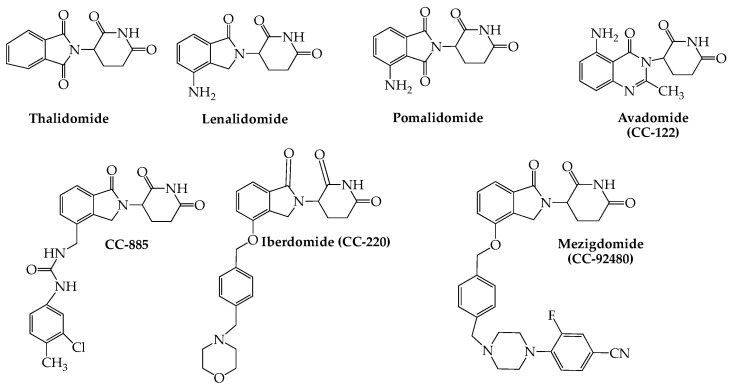
Structures of Immunomodulators (Cereblon E3 Ligase Modulators) With Activity Against MM.

In addition to thalidomide, lenalidomide, and pomalidomide, a number of other small molecule cereblon E3 ligase modulators (CELMoDs) have been developed for treatment of hematologic cancers, including MM. This group of newer oral agents (Figure 2) includes iberdomide (CC-220), which has been shown to bind to cereblon with an affinity that is twenty times higher than that of either lenalidomide or pomalidomide, leading to more efficient degradation of Ikaros and Aiolos (see above) [27]. The combination of iberdomide with dexamethasone in heavily pretreated MM patients yielded ORRs of 32% (29/90; dose-escalation cohort) and 26% (28/107; dose-expansion cohort) with serious adverse events noted in 53% (57/107) of subjects (NCT02773030) [28]. Avadomide (CC-122) is another glutarimide-based cereblon modulator that has broader activity than lenalidomide, possibly due to its capacity to cause deeper and faster kinetics of Aiolos degradation in myeloma cell lines [29]. Preliminary results from a phase I study of avadomide monotherapy in NHL, MM, and solid tumors found the drug to demonstrate favorable pharmacokinetics while having an acceptable tolerability and safety profile (NCT01421524) [30]. Mezigdomide (CC-92480), another promising drug in this category, shows enhanced antitumor immunostimulatory activity in myeloma cell lines, including those resistant to pomalidomide and lenalidomide [31]. This agent in combination with dexamethasone and a proteasome blocker is currently included in two myeloma-based phase III clinical studies comparing its activity both with (NCT05519085) and without (NCT05552976) pomalidomide (see Table 1). Although no data have yet appeared for either of these studies, recent reports of an ongoing phase I/II trial (NCT03374085) employing mezigdomide and dexamethasone showed promising efficacy with manageable toxicity in triple-refractory RRMM patients [32,33].

## 4. Proteasome Inhibitors

Protein turnover in eukaryotic cells is accomplished primarily via the ubiquitin-proteasome pathway, which is initiated by the addition of ubiquitin, a highly conserved 76-residue polypeptide, to an e-amino group on a lysine residue on the protein intended for disposal. Once linked, the ubiquitin moiety itself can be ubiquitinated several times in tandem, thus tagging the protein for delivery to the proteasome. The ubiquitination process actually comprises three separate steps: ATP-dependent activation of E1, conjugation catalyzed by E2, and finally ligation effected by E3 [36]. In contrast to E1 (two are known) and E2 (about 60 have been identified), the human genome codes for several hundred different E3s, one of which (cullin-4 RING E3 ligase or CRL4) was discussed earlier in Section 3.

**Figure 3:**
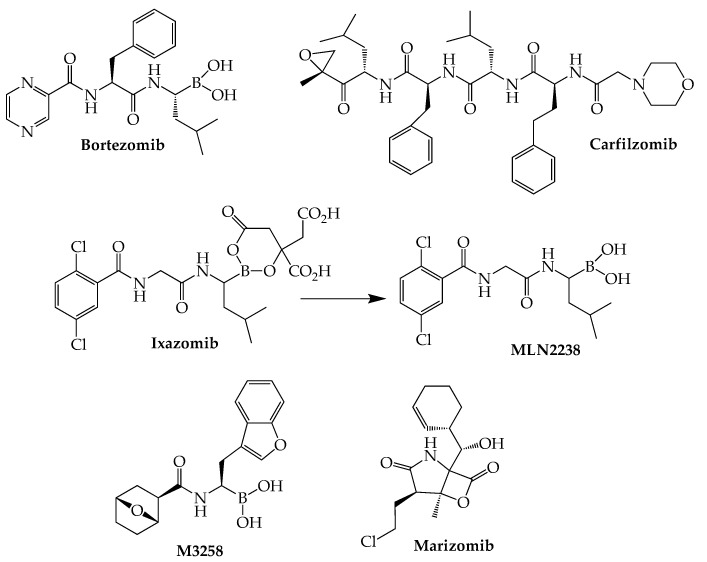
Structures of Proteasome Inhibitors Active Against MM.

The boronic acid dipeptide bortezomib (Velcade), the first proteasome inhibitor to be studied extensively, was approved by the FDA in 2003 for MM, marking a significant turning point in the treatment of this cancer. In comparison with bortezomib, the second-generation proteasome blocker carfilzomib (Kyprolis) has demonstrated a reduced capacity to produce the peripheral neuropathy associated with bortezomib while generating more sustained and deeper patient responses, even in patients who relapse following bortezomib treatment [37]. Carfilzomib’s actions are attributed to its capacity to irreversibly block ChT-L sites as a result of ring opening of its epoxyketone structure. However, use of this agent is hampered by its tendency to produce cardiotoxicity, as demonstrated by two recent network meta-analyses of randomized clinical trials [38,39]. Ixazomib (Ninlaro), which like bortezomib is a boronic acid-derived peptide reversible beta-5 CT-L inhibitor, is the most recently (2015) approved anti-myeloma agent in this class. Compared to its two predecessors, ixazomib is associated with reduced risk of peripheral neuropathy while offering the distinct advantages of oral bioavailability and once-weekly dosing [40]. Acting as a prodrug, ixazomib is rapidly converted to MLN2238, which is responsible for the drug’s pharmacological properties [41] (Figure 3).

The naturally occurring marizomib (salinosporamide A) is a marine-derived proteasome inhibitor that lacks the peptide-like structure common to the other members of this class. The high lipophilicity of this intravenously administered inhibitor of all three types of proteasomes enables its passage through the blood–brain barrier, making it potentially useful in extramedullary cases of myeloma involving the central nervous system. Following demonstration that marizomib synergizes with pomalidomide in myeloma cell cultures [42], a phase I trial (NCT02103335) of the combination together with low-dose dexamethasone gave an ORR of 53% (19/36) in RRMM (median of four prior therapies) [43]. The same combination currently is the subject of a phase II trial (NCT05050305) involving 30 MM patients with CNS involvement. TQB3602 represents another promising anti-myeloma proteasome inhibitor currently in a clinical trial (NCT04275583). Although its full structure has not been disclosed, this oral agent is said to be an acridine-containing derivative of ixazomib. Two preliminary reports recently have appeared describing the safety and efficacy of TQB3602 in RRMM patients [44,45].

## 5. Deubiquitinase Inhibitors

The removal of ubiquitin tags prior to peptide cleavage occurs in the proteasomal 19S RP caps. Mammalian 19S RPs are known to contain three major types of deubiquitinases: ubiquitin-specific peptidases (USPs), ubiquitin C-terminal hydrolases (UCHs), and a zinc metalloprotease. Interest in DUB inhibitors in MM stems from reports that several of these DUBs, especially those of the USP class, are upregulated in MM and are associated with poor prognosis [46]. For example, USP7 overexpression has been linked to bortezomib resistance, and a small molecule inhibitor (P5091) of USP7 has been shown to overcome this resistance and produce apoptosis in myeloma cells [47]. A myeloma-based clinical trial (NCT02372240) of VLX1570, which inhibits both USP14 and UCHL5 and showed promise in MM in preclinical work, had to be terminated due to pulmonary toxicity [48,49].

## 6. Neddylation Inhibitors

Discovery of an alternative pathway for protein degradation, which employs ubiquitin-like proteins (ULPs) and accounts for about 20% of protein loss in proteasomes, also has spurred a search for new targets. One such target is the ULP known as NEDD8 (neural precursor cell expressed developmentally downregulated-8), an 81-amino acid peptide that shares considerable homology (80%) and sequence identity (60%) with ubiquitin. Like ubiquitin, each ULP has its own series of conjugating enzymes (E1–E3). Cullin-RING (real interesting new gene) ligase, also known as NEDD8-activating enzyme (NAE), is a ubiquitin ligase subtype that catalyzes the attachment of NEDD8 (neddylation) in preparation for its linkage to a lysine residue on the target protein, a number of which are enzymes whose overexpression in several cancer types correlates with tumor progression and unfavorable patient outcomes [50,51,52,53,54]. Correlation of poor patient prognosis and elevated NEDD8 transcript levels have been noted in bortezomib-treated MM and provided impetus for the search for NAE inhibitors as potential anti-myeloma agents [55], focusing on two agents: pevonedistat (MLN4924) [56] and TAS4464 [57]. The former, which has been under study for a number of hematologic cancers, primarily MDS, was included as a single agent in a trial (NCT00722488) with RRMM patients, who failed to respond [58]. A second myeloma-based trial (NCT03770260) of pevonedistat in combination with ixazomib recently was suspended for unstated reasons pending a company decision, while an initial trial of TAS4464 (NCT02978235) has been terminated due to drug-induced liver toxicity.

## 7. HDAC Inhibitors

The nucleosome, which comprises the basic organizational unit of chromatin in all eukaryotes, is an octameric structure formed by four pairs of histones (H2A, H2B, H3, and H4) with approximately 146 DNA base pairs coiled around each octameric set. The compactness of this winding is the key feature that determines DNA’s ability to access the cell’s transcriptional machinery. Tight winding (heterochromatin) tends to favor gene silencing, while gene expression is associated with more open conformations (euchromatin). Histone posttranslational modifications, such as acetylation, methylation, phosphorylation, ubiquitination, sumoylation, and ADP ribosylation that occur primarily on lysine residues, play important roles in regulating gene expression [59,60]. These alterations in histone structure, together with DNA-base methylations (primarily at cytosines), constitute “epigenetics”, i.e., changes in genetic expression not due to nucleotide sequence changes. Acetylation of e-amino groups of lysine residues situated at N-termini of histone tails constitutes one of the most important of these histone modifications. By removing the positive charge on these residues, acetylation causes relaxation of the DNA-histone interaction, thereby enhancing DNA accessibility and consequent activation of transcription. Agents that promote the addition or loss of these acetyl groups via histone acetyltransferases (HATs) or histone deacetylases (HDACs), respectively, have occupied an important niche in anticancer drug research since the late 1990s [61]. In addition to core histones, HDACs are known to catalyze removal of acetylated lysines from nonhistone proteins, including p53, HSP90, tubulin, and several of those involved in cell cycle control, angiogenesis, and DNA damage repair, among other processes, resulting in defective gene expression and cellular signaling [62,63].

Four classes of classical HDACs, encompassing 11 canonical subtypes, are known: I (includes HDACs 1–3 and 8); IIA, (HDACs 4, 5, 7, and 9); IIB (HDACs 6 and 10), and IV (HDAC 11). Although members of all four classes are dependent on Zn^+2^ for activity, they differ in sequence homology, substrate specificity, and cellular distribution (Class I: nucleus; Class IIB: cytoplasm; Classes IIA and IV: nucleus and cytoplasm). A Class III, referred to as sirtuin 2 in yeast and sirtuins 1–7 in mammals, differs from the others in that its members are dependent on NAD^+^ for activity [64].

**Figure 4:**
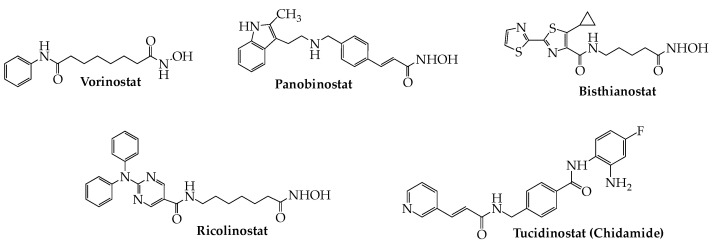
Structures of Histone Deacetylase Inhibitors Active Against MM.

Dysregulation of HDAC has been reported in several solid tumors and in hematological cancers, including MM, and in a number of instances overexpression has been linked to poor prognosis [65,66,67]. As a consequence, inhibitors of HDAC (Figure 4) have received an abundance of attention as potential antitumor drugs [68]. Three inhibitors of all HDAC classes (pan-HDAC inhibitors)—vorinostat, belinostat, romidepsin—received FDA approval for T-cell lymphomas during the 2006–2014 period, but each failed to demonstrate any clear benefit as a single agent in MM. Of the three, only vorinostat has been the subject of several trials in combination with established anti-myeloma agents (Table 2). In the most extensive of these studies reported to date, the combination of vorinostat and bortezomib in RRMM patients produced a median PFS advantage of 0.8 months (7.63 vs 6.83) over bortezomib + placebo, although the relevance of this difference was not clear due to treatment schedule differences between the two cohorts (NCT00773747) [69]. In 2015, another pan-HDAC blocker, panobinostat, was granted accelerated approval for RRMM based on the results of the phase III PANORAMA-1 study (NCT01023308) in which its addition to a bortezomib/dexamethasone regimen provided a PFS benefit of 7.8 months [70]. This approval was accompanied by a required warning regarding the drug’s increased risk of potentially fatal cardiac toxicity and severe diarrhea, as revealed in the PANORAMA-1 trial. However, the drug’s sponsor failed to conduct the required post-approval studies, and in Nov. 2021 the drug’s approval was withdrawn. Additionally, preliminary RRMM patient data have begun to appear for single-agent bisthianostat, another oral pan-HDAC blocker (NCT03618602) [71,72]. In the meantime, second-generation orally active isotype-selective HDAC blockers have risen to a position of prominence. Foremost among these are the HDAC6 inhibitors ricolinostat (ACY-1215) and citarinostat (ACY-241), as well as tucidinostat (chidamide), which targets HDACs 1, 2, 3, and 10 (Table 3). The chemistry of the latter differs from that of most other HDAC inhibitors in that it lacks the zinc-binding hydroxamate group. Results of studies with citarinostat in MM have yet to be reported, but two phase Ib RRMM studies of ricolinostat revealed ORRs, respectively, of 37% when combined with bortezomib/dexamethasone (NCT01323751) [73] and 55% in combination with lenalidomide/dexamethasone (NCT01583283) [74]. However, neither of these trials contained a control arm, making comparison with established regimens difficult. Early results from a small study (*n* = 11) of transplant-ineligible MM patients in which tucidinostat was added to a bortezomib-lenalidomide-dexamethasone (VRd) regimen reported an ORR of 90.9% compared to 100% for VRd alone. Patients in the study group were found to experience more adverse events than those in the VRd control [75].

## 8. Bromodomain Inhibitors

In addition to their major influence on chromatin architecture, lysine acetylation marks on histones are now known to play more complex and diverse roles in transcription regulation. Importantly, transcription factors bearing a bromodomain (BRD) have the capacity to recognize (“read”) and be recruited to these acetylation sites. Bromodomains, highly conserved structural modules of approximately 110 amino acids, facilitate interactions, that often are transient, within transcriptional complexes to impact the epigenetic regulation of gene transcription. The human genome contains 61 different bromodomains arranged in eight subgroups according to structural and sequence similarities [86]. One of these groups, the BRD and extra-terminal (BET) family, is distinguished by the presence of two N-terminal BRDs and includes as its major members BRD2, BRD3, BRD4, and the testis-specific BRDT. In recent years, BRD4 has been identified as the major BRD involved in oncogenesis and consequently has been the focus of several anti-cancer drug discovery efforts [87,88]. Among the cancer-associated proteins whose expression is downregulated by small molecule BRD4 inhibitors are the oncogenic driver c-Myc [89,90], the anti-apoptotic Bcl2 [91], and cell cycle regulators such as the cyclin-dependent kinases [92]. In particular, c-Myc has been shown to be activated in as many as 50% of MM patients [93], and in preclinical work, JQ1, a BRD4 inhibitor, demonstrated anti-myeloma activity associated with cellular senescence and cell cycle arrest [90,94]. However, in a phase I dosing trial that also included lymphoma patients (NCT01713582), birabresib (OTX015) [95], a close structural relative of JQ1, showed no activity in a group of 12 MM patients [96]. Phase I myeloma-based studies of two other members of the BET family—RO6870810 (NCT03068351) and pelabresib (CPI-0610) [97] (NCT02157636)—have been completed. In the former study, a cohort of 24 RRMM patients showed a low response rate with a high rate of thrombocytopenia and anemia over a range of dosages when used as monotherapy [98]. No data have appeared yet concerning responses of RRMM patients to pelabresib, although a preclinical study of this drug indicated potential synergy with lenalidomide [99].

## 9. Apoptosis Inducers: Bcl2, IAP, and Mcl-1 Inhibitors

The capacity of cancer cells to evade apoptosis constitutes one of their major mechanisms of survival. The apoptotic machinery encompasses two basic pathways, extrinsic and intrinsic, each with its own ability to upregulate different sets of mediators that eventuate in cell death. The extrinsic pathway is initiated by external ligands that bind to cell surface death receptors, primarily members of the tumor necrosis factor (TNF) superfamily, resulting in downstream events including activation of caspase-8, a critical mediator of apoptosis. Members of the Bcl2 family of proteins, the primary regulators of the intrinsic pathway, may possess either pro-death or pro-survival properties. Each works by heterodimerizing with members having the opposite attribute, resulting in neutralization. The conserved Bcl2 homology (BH) death domains, numbered BH 1–4, are critical components found in both pro- and anti-apoptotic members. The major pro-apoptotic members are Bax and Bak, which contain only BH3 domains, while Bcl2 itself (the founding member), Bcl-xL (B-cell lymphoma-extra large), and Mcl-1 (myeloid cell leukemia-1) are anti-apoptotic proteins that may contain any one of the four BH domains. In recent years, there has been much interest in discovering small molecule BH3-selective mimetics having the ability to block the interactions between these mutually antagonizing types of proteins to affect cancer cell death [100,101]. The first compound to emerge successfully from these studies was the orally bioavailable venetoclax (Venclexta, ABT-199) [102,103], which initially received FDA approval in 2016 for use in CLL with 17p deletion, since expanded to include acute myeloid leukemia (AML). Currently, there are two phase III studies: NCT02755597 (BELLINI) and NCT03539744 (CANOVA) investigating the potential of venetoclax in RRMM. The recently concluded BELLINI trial demonstrated that addition of venetoclax to a bortezomib-dexamethasone regimen resulted in significant improvement in PFS compared to the cohort receiving placebo (23.4 months vs. 11.4 months). Significantly, the greatest improvement in PFS was seen in patients having either t(11;14) (36.8 months vs. 9.3 months) or high Bcl2 (30.1 vs 9.9 months). However, increased mortality, primarily due to heightened infection rates, in the study group compared to placebo (7% vs. 2%) was a concern [104] and caused temporary holds, later lifted, to be placed on both phase III studies. Other studies, although in earlier phases and smaller enrollments, (Table 4), have tended to substantiate the efficacy of venetoclax in t(11;14) RRMM. Results from the CANOVA trial (venetoclax plus dexamethasone vs. pomalidomide plus dexamethasone), which includes only t(11;14) RRMM patients and a planned patient size of 244, have yet to be reported but should throw important light on the future role of venetoclax in this biomarker-based MM patient group [105]. Meanwhile, lisaftoclax (APG-2575), another Bcl2 inhibitor, recently entered an RRMM trial both as a single agent and in a lenalidomide/dexamethasone combination (NCT04674514) [106]. Furthermore, recruitment recently began for an RRMM study using the highly selective Bcl2 blocker BGB-11417 [107] as both monotherapy and in different combinations with dexamethasone and carfilzomib (NCT04973605). A preliminary safety study has been published on a cohort of 10 t(11;14)-positive RRMM patients in this trial, indicating that the combination of BGB-11417 with dexamethasone generally is well-tolerated [108].

**Figure 5:**
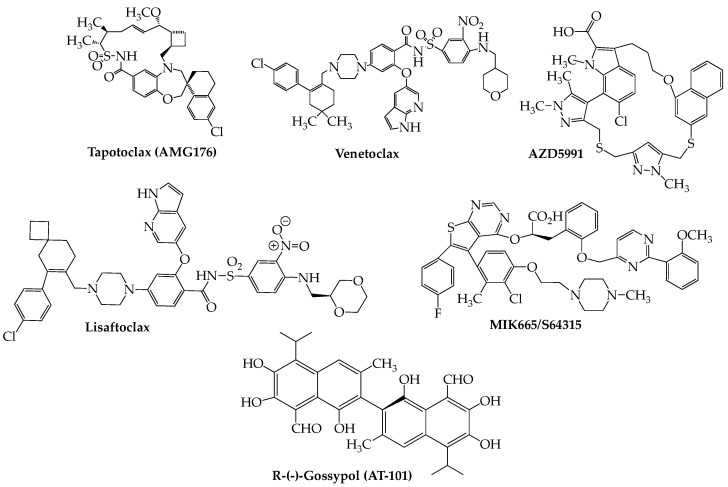
Chemical Structures of Apoptosis Inducers Active Against Myeloma.

The aforementioned anti-apoptotic Mcl-1 recently has emerged as a druggable target for treatment of hematologic cancers [109]. The most promising agent in this space is tapotoclax (AMG176) (Figure 5) (NCT02675452). A preliminary report on 26 RRMM subjects (median of five prior lines of therapy) who received tapotoclax intravenously indicated that 22 patients discontinued therapy due to disease progression and that treatment-related adverse events (hematologic and gastrointestinal) were noted in all but one patient [110]. Other Mcl-1 inhibitors under active consideration for use in RRMM as well as other hematologic cancers include S-64315/MIK665 (NCT02992483) [111], PRT1419 (NCT04543305) [112], and AZD-5991 (NCT03218683) [113].

Note should also be made of the pan-Bcl2 inhibitor R-(-)-gossypol (AT-101), an orally bioavailable polyphenol derived from the cotton plant, which has been investigated in several cancers [114] and is presently under study for RRMM in combination with lenalidomide and dexamethasone (NCT02697344). An initial report on 10 subjects in this trial indicated that a Bcl2 inhibitor-immunomodulator regimen demonstrates clinical activity (ORR = 40%) with an acceptable toxicity profile [115].

For a full discussion of the current status of Bcl2 and Mcl-1 small molecule inhibitors in MM, the reader is referred to the recent review by Parrondo et al. [116]

**Table 4:** Efficacy of Venetoclax in t(11;14) RRMM.

Trial ID (Reference)	Phase	Drugs	Enrollment (N)	Prior Lines of Therapy (Median)	ORR (%)	PFS (Median in Months)
NCT03314181 [117]	I	(Ven + Dara + Dex) vs. (Ven + Dara + Dex + Bort)	Part 1: [24 with t(11;14)—Ven + Dara + Dex]; Part 2: [6 with t(11;14) + 18 other RRMM— Ven + Dara + Dex + Bort]	Part 1: 2.5; Part 2: 1	Ven + Dara + Dex: 96; Ven + Dara + Dex + Bort: 92	NR
NCT03314181 [118]	I/Ii	(Ven + Dara + Dex) vs. (Ven + Dara + Dex + Bort)	34 all t(11;14): 11 Ven + Dara + Dex (12 at 400 mg Ven, 7 at 800 mg. Ven); 16 Ven + Dara + Dex + Bort	Ven + Dara + Dex: 1; Ven + Dara + Dex + Bort: 2	Ven + Dara + Dex: 72.7 (at 400 mg.) and 100 (at 800 mg.); Ven + Dara + Dex + Bort: 62.5	NR
NCT02899052 [119]	II	Ven + Carf + Dex	49: 13 t(11;14); 36 non-t(11;14)	1	t(11;14): 92; non-t(11;14): 75	With t(11;14): 24.8; without t(11;14): 22.8
NCT01794520 [120]	I	Ven and Ven + Dex	66: 30 t(11;14); 36 non-t(11;14)	5	t(11;14): 40; non-t(11;14): 6	t(11;14): 6.6; non-t(11;14): 1.9
NCT01794520 [121]	I/II	Ven + Dex	Phase I: 20; Phase II: 31. All t(11;14) positive	Phase I: 3; Phase II: 5	Phase I: 60; Phase II: 48	Phase I: 12.4; Phase II: 10.8
NCT02755597 [122]	III	(Ven + Bort + Dex) vs. (Bort + Dex + Pbo)	291: 35 with t(11;14); 194 (Ven + Bort + Dex), 97 (Bort + Dex + Pbo)	1–3	NR	With t(11;14): Ven + Bort + Dex: 36.8; Bort + Dex + Pbo: 9.3

Bort = bortezomib; Dara = daratumumab; Dex = dexamethasone; NR = not reached; Pbo= placebo; Ven = venetoclax.

## 10. Kinesin Spindle Protein Inhibitors

**Figure 6:**
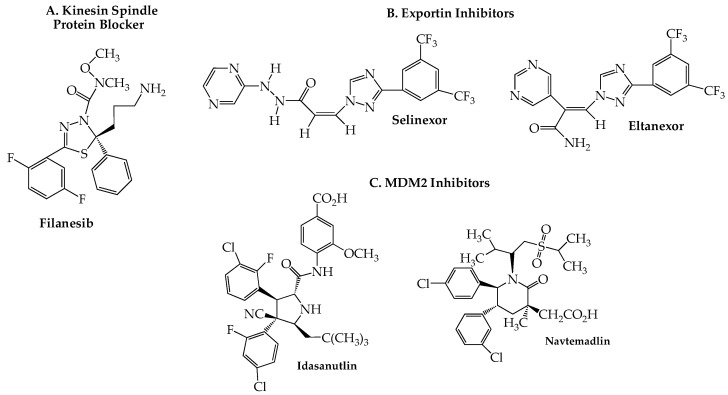
Structural Formulas of Additional Small Molecules with Activity Against MM.

Eg5, a member of the kinesin-5 family of spindle microtubule-associated proteins, plays a key role in cell division by providing the mechanical energy, via ATP hydrolysis, needed for centrosome separation and bipolar spindle assembly during mitosis. Inhibition of Eg5, also known as kinesin spindle protein (KSP), causes formation of a monopolar (instead of the normal bipolar) spindle, resulting in mitotic cell cycle arrest at the spindle checkpoint and consequent apoptosis [123]. Since KSP inhibitors block mitosis without directly affecting microtubules, they may offer an advantage by avoiding the peripheral neurotoxicity seen with antitumor drugs, such as the vinca alkaloids and taxanes, which act directly on microtubules. Preclinical studies uncovered filanesib (ARRY-520) (Figure 6A) as a highly potent and selective KSP inhibitor [124,125]. Encouraging ORR data were obtained in RRMM patients in an initial trial (NCT00821249) with filanesib as a single agent (16%; 5/31) and in combination with dexamethasone (15%; 8/54) [126]. A subsequent study (NCT01248923) reported an ORR of 42% (8/19) in a well-tolerated regimen of filanesib with bortezomib and dexamethasone [127]. Moreover, this latter combination recently has shown encouraging activity in high-risk RRMM patients with the t(11;14) aberration [128]. Especially noteworthy is the efficacy (ORR = 45%) that this combination demonstrated in a subset of 11 patients harboring the 1q21 gain high-risk cytogenetic abnormality, a biomarker that other studies have shown to be associated with poor outcomes with daratumumab and standard triplet regimens [129,130,131]. A trial of another proteasome inhibitor, carfilzomib, with filanesib, and dexamethasone (NCT01372540) was found to produce only marginal clinical benefit (ORR 37%, median PFS 4.8 months, and median OS 24.9 months), although no biomarker-based stratification was included in this phase I unrandomized study [132]. An RRMM-based study (NCT02384083) of filanesib with a pomalidomide/dexamethasone regimen found a median PFS of 7 months with an OS of 19 months and substantial hematological toxicity [133]. Alpha 1-acid glycoprotein (AAG), a protein whose elevation has been found in NDMM patients [134], appears to play an important prognostic role in filanesib therapy, as first reported in a preclinical study [135]. Data reported in several of the trials shown in Table 5 have tended to confirm linkage of high plasma AAG levels to poor clinical responses to the drug, likely due to the ability of plasma AAG to sequester filanesib, effectively reducing its blood concentration to subtherapeutic levels [136].

## 11. Exportin Inhibitors

Transport of RNA and proteins both out of and into the nucleus is controlled by the nuclear pore complex (NPC), a large cylindrical multiprotein complex whose detailed architecture recently has been reported [139,140]. Although small molecules are able to diffuse passively through the NPC, macromolecules (larger than 30–60 kDa) require a nuclear transport receptor to enable nucleocytoplasmic shuttling. Such receptors are members of the karyopherin-b superfamily and are of two major types: exportins and importins. Exportin 1 (XPO1, originally known as chromosomal region maintenance 1), one of the best characterized members of the former group, is responsible for transporting a wide range of proteins (over 200 are known), including tumor suppressor proteins, transcription factors, cell cycle regulators, as well as mRNA transcripts, from the nucleus into the cytoplasm. Overexpression of XPO1 has been associated with poor outcomes in terms of OS and PFS in several cancers [141], including hematologic malignancies such as MM [142,143]. The validity of XPO1 as a therapeutic target for MM was confirmed in a number of reports, including genome-wide studies [144], and led to the discovery and eventual approval of the orally effective XPO1 blocker selinexor (Xpovio) (Figure 6B) for the treatment of MM. Like other members of this drug class, sometimes referred to as selective inhibitors of nuclear export (SINE), selinexor slowly forms a covalent bond with cysteine 528 in the nuclear export cargo-binding pocket of XPO1 [145]. Selinexor was first approved by the FDA in 2019 for use with low-dose dexamethasone for the treatment of RRMM in patients who had received at least four prior therapies [146]. Approval was granted on an accelerated basis predicated on the response rates and toxicity data obtained in the STORM trial (NCT02336815) [147]. The results of a confirmatory trial (NCT03110562; BOSTON) in which selinexor was combined with bortezomib and dexamethasone led in the following year to approval for use in even earlier stages of the disease, i.e., after at least one prior therapy [148,149,150]. In order to lessen the development of severe adverse effects (thrombocytopenia, neutropenia, gastrointestinal and neurological toxicity, hyponatremia), the selinexor-bortezomib-dexamethasone combination is administered on a once-weekly basis. Current trials that include selinexor in RRMM are shown in Table 6. In a phase I study (NCT02649790), eltanexor (KPT-8602) (Figure 6B), a second-generation oral XPO1 blocker, demonstrated improved efficacy and tolerability over selinexor when combined with dexamethasone in RRMM [151].

## 12. MDM2 Blockers

The tumor suppressor gene *TP53* and its protein product p53 have been widely studied for their roles in a wide number of cellular responses to stress, including hypoxia, DNA damage, oncogene activation, cell cycle arrest, and apoptosis [166]. The highly complex actions of p53 appear to be most important in modulating the transcription of hundreds of genes involved in normal cellular homeostasis. Often referred to as “Guardian of the Genome”, *TP53* is found to be mutated in 50–60% of all human cancers [167]. Moreover, p53 also is known to play a role in a number of processes apparently unrelated to its transcriptional activities [168]. Deletion of the *TP53* locus on chromosome 17p is one of the most commonly noted genetic aberrations associated with high-risk MM, being found in 8% of NDMM patients but rising to as high as 45% upon relapse [169].

MDM2 (murine double minute 2) is one of the most widely studied p53-controlling effectors in the cell and is known to regulate p53 through two major mechanisms. First, acting as an E3-ubiquitin-protein ligase, MDM2 promotes p53 ubiquitination, marking it for proteasomal degradation [170]. Second, MDM2 binds to the p53 N-terminal domain, inhibiting p53′s capacity to effect transcription [171]. MM is one of several cancers in which MDM2 is upregulated, making it a prime target for new anti-myeloma drug development [172].

Currently, two oral agents classed as MDM2 inhibitors are in active clinical trials exploring potential synergy with proteasome inhibitors for RRMM: idasanutlin (RG7388, RO 5503781) [173] and navtemadlin (AMG-232, KRT-232) (Figure 6C) [174]. The first is under investigation in a small trial (NCT02633059; *n* = 12) with ixazomib and dexamethasone limited to patients with 17p deletion, while the latter is included in a phase I dose-escalation study (NCT03031730; *n* = 40) in combination with carfilzomib, lenalidomide, and dexamethasone. Although no data have been reported to date for either trial, it should be noted that navtemadlin has been part of a recently completed phase I study that included patients with MM and advanced solid tumors (NCT01723020; N = 107). An initial report found the drug to exhibit an acceptable safety profile, although data on antitumor efficacy was very limited [175].

## 13. Kinase Inhibitors

### 13.1. Bruton’s Tyrosine Kinase Inhibitors

**Figure 7:**
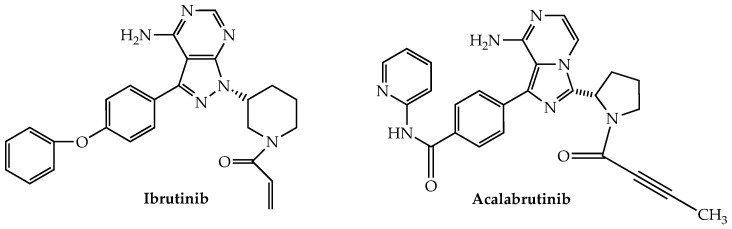
Bruton’s tyrosine kinase inhibitors with anti-myeloma activity.

Bruton’s tyrosine kinase (BTK) is a non-receptor kinase belonging to the TEC family that plays a major role in B-cell development [176]. It also is expressed in T cells and NK cells where it is an important contributor to their activation as well [177,178]. The oral irreversible (by virtue of its covalent binding to Cys-481 in the ATP binding pocket) BTK inhibitor ibrutinib (Figure 7), which has been approved for the treatment of CLL, mantle cell lymphoma (MCL), and Waldenstrom’s macroglobulinemia among others, also has been studied for possible application in MM owing to reports of robust expression of BTK in myeloma cells [179].

In a phase II trial (NCT01478581), ibrutinib provided only modest efficacy when used alone or in combination with dexamethasone in 69 RRMM patients [180]. Combinations of ibrutinib with proteasome inhibitors also have been investigated based on potential synergy noted in preclinical work. Enrollment in one such trial (NCT02902965) that included bortezomib/dexamethasone was suspended and eventually terminated when an increase in serious and fatal infections was noted in the study group, thus making attainment of the study’s target PFS unlikely [181]. In this connection, it is noteworthy that opportunistic infections have been reported as a significant risk in a number of ibrutinib-based studies [182,183]. A phase I/II study (NCT01962792) of ibrutinib plus carfilzomib/dexamethasone showed median PFS and OS of 7.4 months and 35.9 months, respectively, in 59 heavily pretreated (median of three prior treatments) MM patients. While 18 (31%) of the patients in this study experienced upper respiratory infections, most were of grade 1 or 2 and none were fatal [184].

Ibrutinib also has been combined with lenalidomide/dexamethasone in a recently reported phase I dose-escalation study of 15 RRMM subjects who had received a median of four prior therapies (NCT03015792). Initial results reported a median PFS of 3.8 months and, although only one patient attained a partial response (ORR, 7%), clinical benefit as defined by the trial criteria was realized in 12 of the patients (80%). Overall, hematologic adverse effects (≥grade 3) were noted in 20% of the study participants (99675) [185].

Acalabrutinib, a second-generation oral irreversible BTK inhibitor, approved by the FDA for both MCL and CLL, was the subject of a now-completed phase Ib trial for RRMM (NCT02211014). The trial consisted of two arms: acalabrutinib alone (*n* = 13) and with dexamethasone (*n* = 14). No efficacy was demonstrated in either cohort, while serious adverse events were recorded in 38% and 64%, respectively, of the participants.

### 13.2. Transforming Growth Factor Receptor Inhibitors

The transforming growth factor (TGF)-b is a cytokine which effects diverse cellular processes, including growth, differentiation, migration, and cell death. The membrane-bound receptor for TGF-b contains a C-terminal domain possessing serine/threonine kinase activity. Activation of the TGF-b receptor causes phosphorylation of Smads, which in turn translocate to the nucleus where they bind to specific DNA sequences to regulate transcription of target genes [186].

The observation that MM cells demonstrate increased secretion of TGF-b linked to impaired immune surveillance and catabolic bone remodeling [187] led to a phase Ib trial (NCT03143985) of the TGF-b blocker vactosertib and pomalidomide in RRMM. Initial data on 15 patients in this study, conducted without inclusion of steroids, showed that disease progression occurred in only three subjects, while the rest experienced some degree of progression-free benefit. Adverse events were reported as manageable [188].

### 13.3. Raf-Mek-Erk Pathway Inhibitors

The *Ras* gene is known to be the most frequently mutated oncogene in cancer, being found in approximately 19% of all malignancies [189]. The prevalence of *Ras* mutations, primarily as KRAS and NRAS, in NDMM has been estimated in one study as about 46%, rising to 64% in RRMM [190]. Such mutations manifest as increased sequential activation of the three serine/threonine protein kinases that together constitute the Raf/Mek/Erk (MAPK) downstream intracellular signaling pathway. V600E/K mutations of the *Raf* family member BRAF are frequently found in melanoma and other solid tumor types for which the MAPK blocking agents dabrafenib and trametinib, inhibitors of BRAF and Mek, respectively, are used clinically in combination. In addition, this mutation, which is associated with poor prognosis, is found in 2–4% of NDMM patients and about 8% in the RRMM setting [190]. Dabrafenib and trametinib, both in combination and separately, currently are under investigation in RRMM (NCT03091257), as is encorafenib (anti-BRAF) with binimetinib (anti-Mek) (NCT02834364; BIRMA). Although no results have been reported from either study, data are available from another trial that included the Mek inhibitor cobimetinib, which despite lacking single-agent activity, demonstrated potential but limited anti-myeloma efficacy when used together with venetoclax and/or atezolizumab (NCT03312530) [191]. Vemurafanib, another BRAF blocker, whether employed alone [192] or with cobimetinib [193], has been reported to elicit some partial responses in V600E RRMM as recorded in case reports, as well as in a small cohort of patients in another trial (NCT01524978) [194].

### 13.4. PI3K-Akt-mTOR Pathway Inhibitors

Another signaling pathway that operates downstream of Ras, the PI3K-Akt (protein kinase B)-mTOR (mammalian target of rapamycin) route, has received some attention in the search for new targets to treat RRMM but with largely disappointing results [195]. For example, the Akt inhibitor perifosine, which showed initial promise against MM in preclinical and early patient studies, failed to live up to expectations in a subsequent discontinued phase III study [196]. Trials combining the Mek blocker trametinib with Akt inhibitors afuresertib (GSK2110183) (NCT01476137) or uprosertib (GSK2141795) (NCT01951495) generally have yielded modest results [197,198], while data have yet to be reported from an ongoing trial (NCT02144038) of the oral PI3K inhibitor alpelisib (BYL719) with LGH447, a Pim blocker. The major mTOR inhibitors, everolimus and temsirolimus, have fared poorly as single agents in MM trials [199,200], while the few myeloma-based trials that have included mTOR blockers in various combinations heretofore have not produced published results.

## 14. Conclusions

Therapeutic measures to treat MM were solely dependent on alkylating agents and corticosteroids beginning in the mid-1960s. A remarkable transformation in the treatment of the disease began to occur in the 1990s as a result of two landmark discoveries: the beneficial effects of ASCT and the anti-myeloma properties of the once-banned drug thalidomide. Momentum for uncovering novel therapeutic strategies to treat this cancer continued to build with the dawn of the current century and the discovery of the proteasome blocker bortezomib and the thalidomide derivatives lenalidomide and pomalidomide, and more recently, the influx of immunologic-based therapies directed against the myeloma cell-specific surface biomarkers CD38 and BCMA. The cumulative positive impact of these developments is evident when one considers that the five-year survival rate for MM has more than doubled over the past six decades and today exceeds 50%. However, these successes are tempered by the fact that most MM patients become refractory to whatever measure is employed and eventually experience relapse.

This review focused on the continuing search for small molecules, especially those with oral bioavailability, whose addition to the armamentarium of anti-myeloma agents heretofore has proven to be complementary to ASCT and immunotherapies. Particularly noteworthy from the standpoint of new drug discovery are two recent developments that may bear future fruit as platforms for drug design based on artificial intelligence, not only for MM but for other tumors as well. One is the recent atomic-level description of the nuclear pore complex, which controls the cytoplasmic/nuclear transfer of key cancer-linked molecules, such as oncoproteins and tumor suppressor proteins. In this regard, the recently introduced exportin blocking anti-myeloma agent selinexor may portend future advances in which anticancer drugs are exquisitely tailored to affect this critical transport process. The second is the recent upsurge of work in the targeted protein degradation field as noted in Section 3. As far as MM is concerned, while this approach as presently applied to MM targets the transcription factors Ikaros (IKZF1) and Aiolos (IKZF3), it should be applicable in principle to a wide variety of proteins on which initiation and proliferation of MM depend.

The remarkable ability of the small molecules described in this review to elicit reasonably good objective responses and PFS data with manageable toxicity in patients classed both as NDMM and RRMM has been a marked advance in cancer therapy in recent decades. However, the pathway forward poses substantial challenges, as the problem of eventual resistance to therapy will doubtless continue as a major issue. Nevertheless, if the advances made in the past few decades are any indication, the years ahead hold promise for the introduction of new orally efficacious therapeutic options with the capacity to improve the quality of life for patients afflicted with this relentless disease.

## Figures and Tables

**Table 1:** Selected Active Myeloma-based Trials of Cereblon E3 Ligase Modulators.

Trial ID (References)	Drugs	Enrollment (N)	Phase	Trial Title
NCT03374085 [32,33]	CC-92480 vs. (CC-92480 + Dex)	201	I/II	Multicenter, Open-label Study to Assess the Safety, Pharmacokinetics and Efficacy of CC-92480 Monotherapy and in Combination With Dexamethasone in Subjects With RRMM
NCT05519085	(CC-92480 + Bort + Dex) vs. (Pom + Bort + Dex)	760	III	Two-Stage, Randomized, Multicenter, Open-Label Study Comparing CC-92480, Bortezomib and Dexamethasone (480Vd) Versus Pomalidomide, Bortezomib and Dexamethasone (PVd) in Subjects With RRMM (SUCCESSOR-1)
NCT05552976	(CC-92480 + Carf + Dex) vs. (Carf + Dex)	525	III	Two-stage, Randomized, Multicenter, Open-label Study Comparing CC-92480 (BMS-986348), Carfilzomib, and Dexamethasone (480Kd) Versus Carfilzomib and Dexamethasone (Kd) in Participants With RRMM (SUCCESSOR-2)
NCT01421524 [30]	CC-122	271	I	Multi-center, Open-label, Dose Finding Study to Assess the Safety, Tolerability, Pharmacokinetics and Preliminary Efficacy of the Pleiotropic Pathway Modifier CC-122 Administered Orally to Subjects With Advanced Solid Tumors, NHL, or MM
NCT05177536	Iber	38	II	Iberdomide Maintenance Therapy Following ASCT in Patients With MM
NCT04998786	Iber + Ixaz + Dex	80	II	A Multi-center Open-label Study of Ixazomib, Iberdomide and Dexamethasone in Elderly Patients With MM at First Relapse
NCT04975997 [34]	(Iber + Dara + Dex) vs. (Dara + Bort + Dex)	864	III	Two-Stage, Randomized, Multicenter, Open-label Study Comparing Iberdomide, Daratumumab and Dexamethasone (IberDd) Versus Daratumumab, Bortezomib, and Dexamethasone (DVd) in Subjects With RRMM (EXCALIBER)
NCT02773030 [28,35]	Iber vs. (Iber + Dex) vs. [Iber + Dex + (Dara or Bort or Carf)]	449	I/II	Multicenter, Open-label, Dose-escalation Study to Determine the Maximum Tolerated Dose, Assess the Safety, Tolerability, Pharmacokinetics and Efficacy of CC-220 as Monotherapy and in Combination With Other Treatments in Subjects With MM
NCT05392946	Iber + Dara + Bort + Dex	18	I/II	Iberdomide in Combination With Daratumumab, Bortezomib and Dexamethasone in Patients With NDMM (IDEAL)
NCT05199311	Iber + Carf + Dex	66	I/II	Carfilzomib, Iberdomide and Dexamethasone (KID) in Patients With Newly Diagnosed Transplant Eligible MM
NCT04564703	Iber	160	II	Iberdomide Maintenance After ASCT in NDMM Patients
NCT04392037	Iber + Ctx + Dex	60	II	Study of Iberdomide Combined With Low-dose Cyclophosphamide and Dexamethasone in RRMM (ICON)
NCT05558319	(Bort + Isa + Dex +/− Len) vs. (Bort + Isa + Dex + Iber)	480	III	Trial for NDMM Patients Who Are Candidates for ASCT Comparing Extended VRD Plus Early Rescue Intervention vs. Isatuximab-VRD vs. Isatuximab-V-Iberdomide-D

ASCT = autologous stem cell transplantation; Bort = bortezomib; Carf = carfilzomib; Ctx = cyclophosphamide; Dara = daratumumab; Dex = dexamethasone; Iber = iberdomide; Ixaz = ixazomib; NDMM = newly diagnosed multiple myeloma; NHL = non-Hodgkin’s lymphoma; Pom = pomalidomide.

**Table 2:** Selected Myeloma-based Trials of Vorinostat.

Trial ID (References)	Drugs	Enrollment (N)	Phase	Trial Title
NCT00729118 [76]	Vor + Len	19	I	Vorinostat (SAHA) and Lenalidomide After Autologous Transplant for Patients With MM
NCT01502085	Vor + Len + Dex	25	I/II	Vorinostat in Combination With Lenalidomide and Dexamethasone in MM Patients Refractory to Previous Lenalidomide Containing Regimens
NCT00642954	Vor + Len + Dex	31	I	Vorinostat in Combination With Lenalidomide and Dexamethasone in Patients With RRMM
NCT00773747 [69]	(Vor + Bort) vs. (Bort + placebo)	637	III	An International, Multicenter, Randomized, Double-Blind Study of Vorinostat (MK-0683) or Placebo in Combination With Bortezomib in Patients With MM (VANTAGE 088)
NCT00773838 [77]	Vor + Bort + Dex	143	II	An International, Multicenter, Open-Label Study of Vorinostat (MK0683) in Combination With Bortezomib in Patients With RRMM
NCT01720875 [78]	Vor + Bort + Dex	16	II	Trial of Combination Treatment With Vorinostat, Bortezomib and Dexamethasone in Patients With RRMM
NCT01038388 [79]	Vor + Len + Bort + Dex	30	I	Trial Evaluating the Safety and Efficacy of Vorinostat + RVD (Lenalidomide + Bortezomib + Dexamethasone) for Patients With NDMM
NCT01297764 [80]	Vor + Carf + Len + Dex	17	I/II	Study of Carfilzomib, Lenalidomide, Vorinostat, and Dexamethasone in RRMM
NCT01394354 [81]	Vor + Bort + Dex + Doxo	34	I/II	Safety of Vorinostat in Combination With Bortezomib, Doxorubicin and Dexamethasone (VBDD) in Patients with RRMM

Bort = bortezomib; Carf = carfilzomib; Dex = dexamethasone; Doxo = doxorubicin; Len = lenalidomide; Vor = vorinostat.

**Table 3:** Selected Myeloma-based Trials of Isotype-selective HDAC Inhibitors.

Trial Identifier (References)	Drugs	Enrollment (N)	Phase	Trial Title
NCT01323751 [73]	Ricol + Bort + Dex	120	I/II	Open-Label, Multicenter Study of ACY-1215 Administered Orally as Monotherapy and in Combination With Bortezomib and Dexamethasone for the Treatment of RRMM.
NCT01583283 [74]	Ricol + Len + Dex	38	I	Open-Label, Multicenter Study of ACY-1215 (Ricolinostat) in Combination With Lenalidomide and Dexamethasone for the Treatment of RRMM
NCT01997840 [73]	Ricol + Pom + Dex	103	I/II	Multi-Center, Open Label, Dose-Escalation Study to Determine the Maximum Tolerated Dose, Safety, and Efficacy of ACY-1215 (RICOLINOSTAT) in Combination With Pomalidomide and Low-Dose Dexamethasone in Patients With RRMM.
NCT02400242 [82,83,84]	Citar + Pom + Dex	85	I	Multicenter, Single-Arm, Open-Label, Dose-Escalation Study to Determine the Maximum Tolerated Dose, Safety, and Preliminary Activity of Oral ACY-241 Alone and in Combination With Pomalidomide and Low-Dose Dexamethasone in Patients With RRMM
NCT04025450 [75,85]	(Bort + Len + Dex) +/− Chid	50	I/II	Comparation of Chidamide Plus VRD (Bortezomib, Lenalidomide, Dexamethasone) With VRD Regimen for Primary High-Risk MM Patients, a Multiple Center, Randomized Clinical Trial.
NCT03605056	Chid + Len + Dex	25	II	Chidamide in Combination With Lenalidomide and Dexamethasone for the Treatment of RRMM

Bort = bortezomib; Chid = chidamide; Citar = citarinostat; Dex = dexamethasone; Len =- lenalidomide; Pom = pomalidomide; Ricol = ricolinostat.

**Table 5:** Clinical Trials of Filanesib in RRMM.

Trial ID (Reference)	Drugs	Enrollment (N)	Phase	Trial Title
NCT00821249 [126]	Fil vs (Fil + Dex)	55	I/II	A Study of ARRY-520 in Patients With RRMM
NCT01248923 [127,128]	(Fil + Bort) vs. (Fil + Bort + Dex)	55	I	A Study of ARRY-520 and Bortezomib Plus Dexamethasone in Patients With RRMM
NCT01372540 [132]	Carf + Fil	76	I	Filanesib and Carfilzomib in Treating Patients With RRMM or Plasma Cell Leukemia
NCT02384083 [133]	Fil + Pom + Dex	47	I/II	Filanesib (ARRY-520) in Combination With Pomalidomide and Dexamethasone for RRMM Patients
NCT01989325 [137]	(Fil + Carf + Dex) vs. (Carf + Dex)	77	II	A Study of Filanesib (ARRY-520) and Carfilzomib in Patients With Advanced MM
NCT02092922 [138]	Fil	154	II	Trial of Filanesib in RRMM (AfFIRM)

Bort = bortezomib; Carf = carfilzomib; Dex = dexamethasone; Fil = filanesib; Pom = pomalidomide.

**Table 6:** Selected Clinical Trials of Selinexor in MM.

Trial ID (Reference)	Drugs	Enrollment (N)	Phase	Trial Title
NCT02186834 [152]	Sel + Dox + Dex	28	I/II	Investigator-Initiated Trial of Selinexor and Liposomal Doxorubicin for RRMM
NCT02336815 [147,153,154]	Sel + Dex	202	II	Open-Label, Single-Arm Study of Selinexor Plus Low-Dose Dexamethasone (Sd) in Patients With MM Previously Treated With Lenalidomide, Pomalidomide, Bortezomib, Carfilzomib, and Daratumumab, and Refractory to Prior Treatment With Glucocorticoids, an Immunomodulatory Agent, a Proteasome Inhibitor, and Daratumumab (STORM)
NCT05422027	Sel + Len + Bort + Dex	42	I/II	Selinexor Plus Bortezomib, Lenalidomide and Dexamethasone (XVRd) in High Risk NDMM
NCT04661137 [155,156]	Sel + Dex + (Pom or Carf or Dara)	96	II	Study of Selinexor in Combination With Carfilzomib, Daratumumab or Pomalidomide in Patients With RRMM
NCT04414475	(Sel + Dex) vs. (Sel + Dex + Bort)	134	II	Open-label, Multi-arm Trial of Selinexor Plus Low-dose Dexamethasone (Sd) in Patients With Penta-refractory MM or Selinexor and Bortezomib Plus Low-dose Dexamethasone (SVd) in Patients With Triple-class Refractory MM
NCT02780609 [157]	Sel + Mel + Dex	22	I/II	Investigator Sponsored Study of Selinexor in Combination With High-Dose Melphalan Before ASCT for MM
NCT02199665 [158]	Sel + Carf + Dex	100	I	Study of the Combination of Selinexor With Carfilzomib and Dexamethasone in Patients With RRMM
NCT03110562 [149,150,158,159,160]	(Sel + Bort + Dex) vs. (Bort + Dex)	402	III	Randomized, Controlled, Open-label Study of Selinexor, Bortezomib, and Dexamethasone (SVd) Versus Bortezomib and Dexamethasone (Vd) in Patients With RRMM (BOSTON)
NCT05028348	(Sel + Pom + Dex) vs. (Elo + Pom + Dex)	300	III	Randomized, Open-label Trial of Selinexor, Pomalidomide, and Dexamethasone (SPd) Versus Elotuzumab, Pomalidomide, and Dexamethasone (EloPd) in Patients With RRMM
NCT04764942 [161]	(Sel + Pom + Dex +/- Carf	81	I/II	Trial of Selinexor in Combination With Pomalidomide and Dexamethasone ± Carfilzomib for Patients With Proteasome-Inhibitor and Immunomodulatory Drug RRMM (SCOPE)
NCT02343042 [155,162]	Sel + Various	518	I/II	Study of Selinexor in Combination With Backbone Treatments for RRMM and NDMM
NCT03589222 [163]	Sel + Dara + Bort + Dex	62	II	Open-label, Multicenter Trial of Selinexor, Bortezomib and Low-dose Dexamethasone Plus Daratumumab (SELIBORDARA) for the Treatment of Patients With RRMM
NCT04756401	Sel + Dara + Carf + Dex	52	II	Open Label Single-Arm Study of Selinexor, Daratumumab, Carfilzomib and Dexamethasone for High-Risk, RRMM Patients Who Have Received 1–3 Prior Lines of Therapy
NCT04877275 [164]	(Sel + Dex + Dox) vs. (Sel + Ctx + Dex)	50	II	Selinexor in Combination With Chemotherapy to Treat RRMM Relapsed/Refractory Multiple Myeloma Patients
NCT04782687	Sel + Dex + Dara + Len	100	II	Trial of Daratumumab, Lenalidomide and Dexamethasone (DRd) in Combination With Selinexor for Patients With NDMM
NCT04941937	(Sel + Thal + Len) vs. (Sel + Len + Dex) vs. (Sel + Pom + Dex)	90	II	Selinexor in Combination With Immunomodulator to Treat RRMM Patients
NCT04717700	(Sel + Bort + Len + Dex) vs. (Bort + Len + Dex)	100	II	Selinexor With Alternating Bortezomib or Lenalidomide Plus Dexamethasone in Transplant Ineligible NDMM Patients (SABLe): An Investigator Sponsored Trial
NCT04891744	Sel + Thal + Dex	48	I/II	Study of Selinexor in Combination With Thalidomide and Dexamethasone for RRMM
NCT03944057 [165]	Sel + Dex	82	II	Open-Label, Single-Arm Study of Selinexor Plus Dexamethasone in Patients With MM Refractory to Prior Treatment With Immunomodulatory Agents and Proteasome Inhibitor
NCT04939142	(Bort + Dex) vs. (Sel + Bort + Dex)	150	III	Randomized, Controlled, Multicenter, Open-label Study of Selinexor, Bortezomib, and Dexamethasone (SVd) Versus Bortezomib and Dexamethasone (Vd) in Patients With RRMM
NCT05478993	Sel + Pom + Dex	21	II	Study of Selinexor, Pomalidomide, and Dexamethasone For MM With Central Nervous System Involvement

Bort = bortezomib; Dara = daratumumab; Carf = carfilzomib; Ctx = cyclophosphamide; Dex = dexamethasone; Dox = doxorubicin; Elo = elotuzumab; Len = lenalidomide; Mel = melphalan; NDMM = newly diagnosed multiple myeloma; Pom = pomalidomide; Sel = selinexor; Thal = thalidomide; RRMM = relapsed and/or refractory multiple myeloma.

## Data Availability

Not applicable.
